# Inhibition of Mesothelin as a Novel Strategy for Targeting Cancer Cells

**DOI:** 10.1371/journal.pone.0033214

**Published:** 2012-04-02

**Authors:** Kun Wang, Vidya Bodempudi, Zhengian Liu, Emma Borrego-Diaz, Farnaz Yamoutpoor, Anna Meyer, Richard A. Woo, Weihong Pan, Arkadiusz Z. Dudek, Mojtaba S. Olyaee, Tuba Esfandyari, Faris Farassati

**Affiliations:** 1 Molecular Medicine Laboratory, Divisions of Gastroenterology and Hematology/Oncology, Department of Medicine, The University of Kansas Medical Center, Kansas City, Kansas, United States of America; 2 Department of Medicine, The University of Minnesota, Minneapolis, Minnesota, United States of America; 3 Southern Illinois University Medical Center, Springfield, Illinois, United States of America; University of Pennsylvania School of Medicine, United States of America

## Abstract

Mesothelin, a differentiation antigen present in a series of malignancies such as mesothelioma, ovarian, lung and pancreatic cancer, has been studied as a marker for diagnosis and a target for immunotherapy. We, however, were interested in evaluating the effects of direct targeting of Mesothelin on the viability of cancer cells as the first step towards developing a novel therapeutic strategy. We report here that gene specific silencing for Mesothelin by distinct methods (siRNA and microRNA) decreased viability of cancer cells from different origins such as mesothelioma (H2373), ovarian cancer (Skov3 and Ovcar-5) and pancreatic cancer (Miapaca2 and Panc-1). Additionally, the invasiveness of cancer cells was also significantly decreased upon such treatment. We then investigated pro-oncogenic signaling characteristics of cells upon mesothelin-silencing which revealed a significant decrease in phospho-ERK1 and PI3K/AKT activity. The molecular mechanism of reduced invasiveness was connected to the reduced expression of β-Catenin, an important marker of EMT (epithelial-mesenchymal transition). Ero1, a protein involved in clearing unfolded proteins and a member of the ER-Stress (endoplasmic reticulum-stress) pathway was also markedly reduced. Furthermore, Mesothelin silencing caused a significant increase in fraction of cancer cells in S-phase. In next step, treatment of ovarian cancer cells (OVca429) with a lentivirus expressing anti-mesothelin microRNA resulted in significant loss of viability, invasiveness, and morphological alterations. Therefore, we propose the inhibition of Mesothelin as a potential novel strategy for targeting human malignancies.

## Introduction

Mesothelin (MSLN), a plasma membrane differentiation antigen, is expressed at significantly high levels in several human cancers, including nearly all mesotheliomas [1] and pancreatic adenocarcinomas [2], [3] as wells as about 70% of ovarian cancers [4], [5] and 50% of lung adenocarcinomas [6], [7]. MSLN is detected in over 70% of fine needle aspirates (FNA) of pancreatic adenocarcinomas [2]. Another recent study showed pleural effusion MSLN as a useful marker for detection of malignant pleural mesothelioma [8]. MSLN is also expressed in trace amounts in normal mesothelial cells. *MSLN* gene encodes a 69-kDa polypeptide containing hydrophobic sequence at the carboxyl end which is removed and replaced by phosphatidylinositol. MSLN gene contains 17 exons on human chromosome 16p13.3 and the MSLN cDNA is 2138-bp long, with an open reading frame of 1884 base pair.

Mutant mice with inactivation of both copies of MSLN gene were generated with the purpose of studying the function of this protein although no detectable abnormalities were reported for this phenotype [9] http://clincancerres.aacrjournals.org/cgi/content/full/10/12/3937 - B8#B8. Another set of studies have introduced MSLN to be involved in adhesion since NIH3T3 cells transfected with a MSLN expression vector were more difficult to remove from the culture dishes than non-transfected cells [1]. The possibility of a role for MSLN in adhesion is supported by a study showing that MSLN binds to CA125(MUC16), a member of the mucin family glycoproteins, and that such interaction mediates cell adhesion [4]. Based on these findings, the authors suggested that there may be an important role for CA125 and MSLN in the metastatic spread of cancer [4]. Also, mesothelin interaction with MUC16 was suggested to facilitate peritoneal metastasis [10].

In models such as ovarian cancer, analyses of correlation between MSLN expression, pathological variability and clinical outcomes indicated that high MSLN expression was positively associated with chemo-resistance in epithelial ovarian carcinoma patients and short patient survival time [12]. MSLN and another marker HE4 have been recently studied for their value as markers for detection of ovarian carcinoma [5], [11]. From other malignancies the homologous to MSLN gene, namely *Erc* was found to be over-expressed in rat renal carcinoma [12], [13]. In gastric cancer patients, the MSLN positive group had significantly more nodal involvement and significantly deeper tumor invasion than the MSLN negative group [14]. Interestingly, the 5-year survival rate was found to be higher in MSLN positive group in this study.

Several studies have indicated important interactions between signaling pathways involved in development of malignant phenotype and MSLN. For example, MSLN was found to induce expression of matrix metalloproteinases 7 (MMP-7) [15] or to enhance expression levels of interleukin 6 (IL-6) [16]. Expression of mesothelin is also claimed to confer resistance to apoptosis in response to tumor necrosis factor alpha (TNF-alpha) [17]. The MSLN gene is differentially regulated by members of the Wnt signal transduction pathway [18]. Also, in C57MG mouse mammary epithelial cells, MSLN was up-regulated by Wnt-1. Interestingly, tumors with constitutive activation of the Wnt signaling pathway, such as ovarian and pancreatic cancers, have high MSLN expression. Additional studies are needed to fully define MSLN function as well as the role of MSLN in carcinogenesis. The very limited distribution of MSLN on normal tissues portrays MSLN a suitable candidate for tumor-specific therapy. Although strategies such as using monoclonal antibodies targeted against MSLN have been tried before [19], [20], [21], [22], [23], [24], the effect of direct inhibition of MSLN on the viability of cancer cells remains to be investigated. In addition to the translational ramifications of such investigations, the information obtained is useful for evaluating the role of MSLN in cancer biology. In this work, we studied the effects of silencing MSLN on viability, invasiveness and cell signaling pathways in cancer cells derived from mesothelioma, pancreatic and ovarian cancer. Furthermore, silencing MSLN by a lentivirus expressing anti-mesothelin microRNA (miRNA) was also found to significantly reduce the viability and invasiveness of ovarian cancer cells. We have also investigated the outcome of silencing MSLN on cell signaling characteristics of cancer cells and cell cycle progression in order to further understand the pathways involved in the biological role of this antigen.

## Materials and Methods

### Cell Lines and chemicals

Bxpc3, H2373, Ovcar5, and Skov3 cells (all obtained from the American tissue culture collection, www.atcc.gov other than H2373 which was obtained from national cancer institute, NCI) were cultured in RPMI-1640 medium that was supplemented with 10% fetal bovine serum (FBS) (Sigma, St Louis, MO). Ovcar3 is cultured in RPMI-1640 with 10% FBS with addition of 2 mM L-glutamine and 10 µg/ml insulin. NIH3T3, Panc1, Maipaca2 (from ATCC), and OVca429 cells [25] were cultured in Dulbecco's Modification of Eagle's Medium (DMEM) (Sigma) supplemented with 10% FBS. Huvec cells were cultured in F-12K Medium supplemented with 0.1 mg/ml heparin, 0.05 mg/ml endothelial cell growth supplement, and 10% FBS. HT1080 cells were cultured in MEME supplemented with 10% FBS. The above mediums were supplemented with penicillin (100 IU/ml) and streptomycin (100 mg/ml) (Sigma). 293FT cells were cultured in DMEM (high glucose) supplemented with 10% FBS, 0.1 mM MEM non-essential amino acid, 1 mM L-glutamine, and 1 mM MEM sodium pyruvate. HT1080, NIH3T3, and Huvec cells were purchased from ATCC (Manassas, VA). 297FT cells were purchased from Invitrogen (Carlsbad, CA).

Anti-mesothelin siRNA was ordered from Qiagen (Valencia, CA). It was synthesized in double-stranded format with Alexa Fluor 488(AF488) conjugated to the 3′ end of its sense strand. The siRNA oligo was re-suspended in the provided buffer at final stock concentration of 20 µM.

### SiRNA electroporation

10^7^ to 5×10^7^ cells were re-suspended in 270 µl of Opti-MEM and mixed with 30 µl of 20 µM siRNA stock solution and electroporated (∼240 V, one pulse for 20 milliseconds), so the final concentration of anti-mesothelin siRNA was 2 µM. The brightest cells, i.e., the cells with the most amount of siRNA, were selected on the basis of the presence of Alexa Fluor 488 tag at the 3′ end of siRNA molecule using FACS.

### Cell proliferation assay

Cell proliferation assay was performed using WST-1 kit from Millipore (Billerica, MA) according to the manufacturer's protocol. Briefly, 2,000 cells (sorted by FACS) were plated in each well of a 96-well microplate in a final volume of 100 µl. Then 10 µl/well of WST-1 reagent was added and the plate was incubated for 1 hour in standard culture conditions. During incubation, viable cells convert WST-1 reagent into formazan dye by cellular mitochondrial dehydrogenase. Following this incubation, the absorbance was measured at 440/600 nm.

### Cell invasion assay

The matrigel invasion chambers and falcon companion tissue culture plate were obtained from BD Biosciences (San Jose, CA). For siRNA experiments control and anti-mesothelin siRNA-treated cells (30,000 cells/well) were plated in 24-well plate and incubated at 37°C with 5% CO_2_. Forty-eight hours later, the cells were fixed in 100% methanol and stained in 0.05% crystal violet and photographed to visually count the number of invaded cells. For lentivirus related experiments cells were pre-treated with lentivirus for 3 days and then introduced to the Boyden chamber assay for ∼21 hrs.

### Western blot analysis

Cells were lysed at 5, 24, and 48 hours post-electroporation with 1× cell lysis buffer. Thirty microgram of proteins for each sample was loaded onto 4–20% SDS polyacrylamide gel (Bio-Rad, CA). Proteins were then transferred onto a nitrocellulose membrane. The membrane was blocked, washed, and incubated with the different primary antibodies such as mesothelin (Abcam, MA and LS Bio, WA) and β-actin (Cell Signaling Technology, MA), followed by the HRP-conjugated secondary antibody. After a thorough washing, the blot was exposed to ECL (GE Healthcare, NJ) and autoradiography.

### Cell cycle assay

Cell cycle assay was performed according to manufacturer's instructions. Briefly, about 10^6^ cells were washed twice using CycleTEST PLUS Buffer solution (BD Biosciences, Cat. 340242). Cells were re-suspended in 1 ml of the same buffer. To stain the cells, 250 µl of Solution A and 200 µl Solution B were added and incubated for 10 minutes at RT. Cold Solution C (200 µl) was added and incubated at 4°C for 10 minutes. The cells were filtered through a 35 µm cell strainer and analyzed by FACSort flow cytometer.

### Construction of vector expressing miRNA

We designed and synthesized three miRNA mimics targeting the full length of human mesothelin gene (gene access number: NM_005823.4). Each designed miRNA mimic was 64 nucleotides in length, including partial flanking sequence, the miRNA hairpin, and mature miRNA (italicized/underlined) derived from target gene as follows: 1-TGCTG*ATAGCAGCAGGTCCAATGGGA* GTTTTGGCCACTGACTGACTCCCATT GCCTGCTGCTAT; 2-TGCTG*TTCATGTTCTGGAAAGCAAGG*GTTTTGGCCACTGACTGACCCTTGCT TCAGAACATGAA; and 3-TGCTG*TTTACTGAGCGCGAGTTCTCT*GTTTTGGCCACTGACTGACAGAGA ACTCGCTCAGTAAA.

Using the BLOCK-iT Pol II miR RNAi expression vector kit (Invitrogen, CA), we annealed and cloned the oligos encoding the engineered pre-miRNA into the cloning site (ACGA and CAGG) of pcDNA 6.2-GW/EmGFP-miR vectors that is flanked on either side to allow directional cloning or proper process of pre-miRNA. The pre-miRNA was inserted into the 3′-UTR of EmGFP gene-driven by Pol II promoters. EmGFP allows tracking expression of the miRNA, providing a strong correlation between EmGFP and miRNA expression. Each plasmid was sequenced to confirm the inserted double stranded miRNA oligos. The expressing plasmids were electroporated into Ovcar5 cells or Skov3 cells, and analyzed by FACS to ensure the proper expression of miRNA in ovarian cancer cells.

### Generation of lentiviral particles expressing miRNA

The entry clone was generated by combining pMSLNmiR3 with pDONR 221 construct using BP Clonase II enzyme. The miRNA cassette was transferred into the pLenti6.3/TO/V5-DEST vector containing attR1-attR2 sites using LR Clonase II to create the final lentiviral vector MSLNmiR3. In addition, we created a lentiviral vector encoding scrambled miRNA (Negative Control) (TGCTGAAATGTACTGCGCGTGGAGACCTTTTGGCCACTGACTGACGTCTCCAC GCGCAGTACATTT) (Invitrogen) that does not target any known human gene. The expression of miRNA is driven by CMV promoter. The inserted sequences of miR3 and scrambled miRNA were confirmed by sequence analysis.

### Production, titration and infection of lentiviral particles

The MSLNmiR3 or Negative Control lentivirus was transfected with ViraPower packing mix (Invitrogen, CA) into human 293FT cells using Lipofectamine 2000 in Opti-MEM I medium (Invitrogen, CA) and cultured overnight. The cells were placed under blasticidin (10 µg/ml) selection for 72 hours. The supernatant was collected and lentiviral particles were concentrated using Lenti-X Concentrator (Clontech, CA). Lentiviral stock was diluted in ten-fold serial to infect the HT1080 cells. Forty-eight hours post-infection, the cells were assessed by FACS and the titration was calculated using the formula [FxC/V]xD, where “F” is the frequency of GFP-positive cells; “C” is the total number of cells in the well at the time of transduction; “V” is the volume of inoculums in mL; and “D” is lentivirus dilution. Ovca429 cells were infected with lentivirus particles at MOI∼30 in the presence of polybrene (10 µg/ml) overnight. The cell proliferation assay was performed post-infection at the indicated time points.

### Flow cytometry

Cultured cells were dissociated with cell dissociation buffer (Sigma-Aldrich, MO) and washed twice in MACS buffer (PBS plus EDTA and 0.5% bovine serum albumin) (Miltenyi Biotec, CA). A total of 2×10^5^ cells (100 µl) were incubated with mesothelin monoclonal antibody (final concentration, 1 µg/ml) (Cat#ab3362, Abcam, MA) on ice for 1 hour in the darkness. Cells were then washed twice with MACS buffer, re-suspended in 100 µl of a secondary antibody (1∶200 dilution, APC conjugated IgG; BD Biosciences, NJ), and incubated on ice for 1 hour in the darkness. Cells were washed twice and analyzed on LSRII analyzer (BD Biosciences, NJ) using the software FACS Diva version 6.1. Cells stained with secondary antibody alone were included to prove the specificity of antibody.

## Results and Discussion

**Figure 1:**
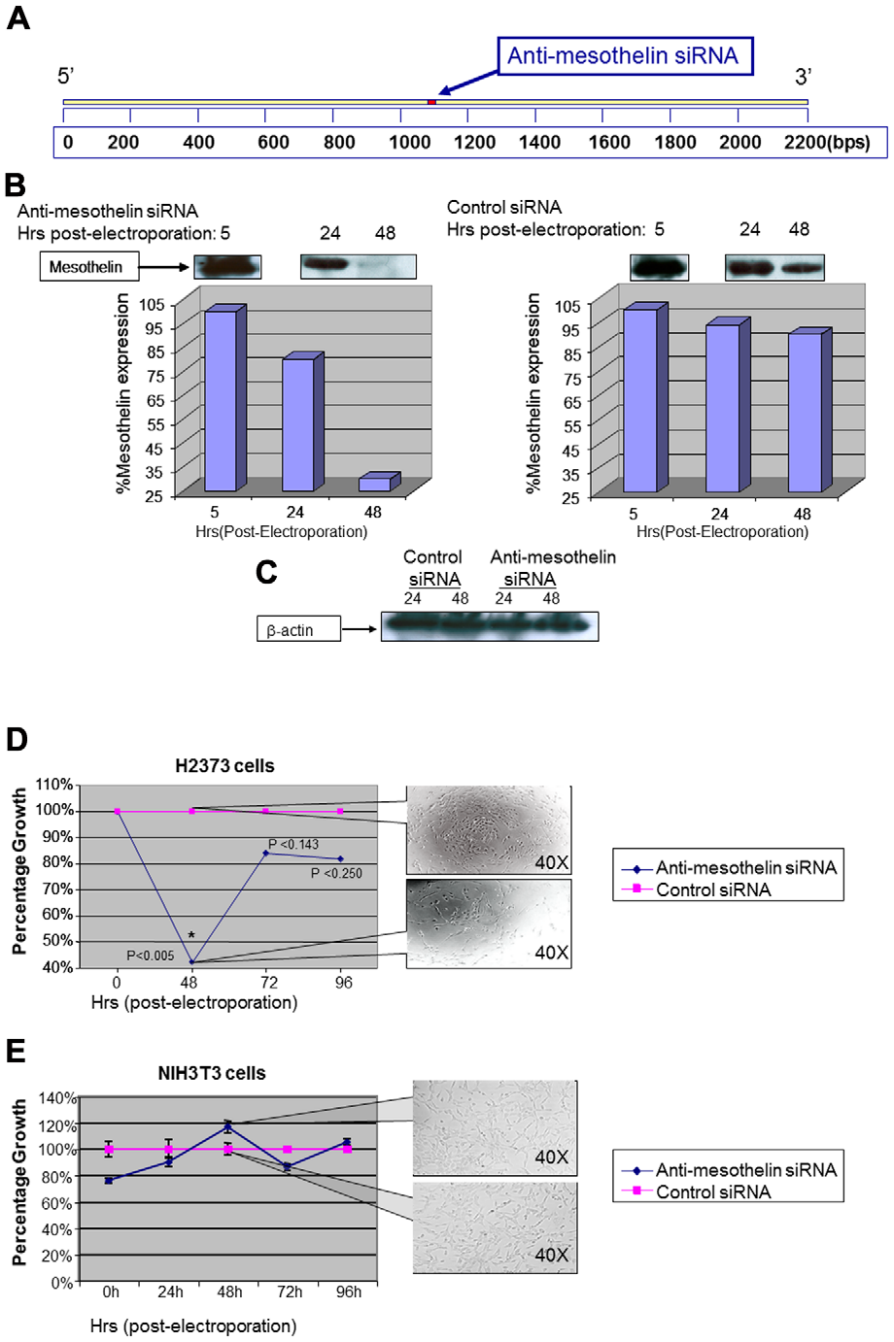
Gene specific silencing of mesothelin reduces proliferation of mesothelioma cells. (A)Anti-mesothelin siRNA was designed to a middle sequence position in mesothelin mRNA. (B)Once electroporated with anti-mesothelin siRNA, the expression levels of mesothelin was significantly reduced in H2373 cells. Negative control siRNA did not cause such reduction. Lower panel shows the results of band-densitometry comparing the intensity of mesothelin expression upon electroporation of H2373 cells with siRNA. (C)Anti-mesothelin siRNA did not affect the expression levels of β-actin, a house-keeping protein, as an evidence for the specificity of this anti-mesothelin siRNA for its target. (D) Proliferation rate of H2373 cells is significantly (p<0.05) reduced at 48 hours post-electroporation to 40% of the values for negative control treated cells. A rebound to higher proliferation rates is observed due to clearance of siRNA from cells at later time points in harmony with our previous studies. Callout panels show the density of cells in each group of the study at 48 hours post-electroporation. (E) NIH3T3 cells are void of mesothelin and their proliferation rate is not affected by exposure to anti-mesothelin siRNA (mouse). Callout panels show the density of cells at 48 hour post-electroporation.

We decided to evaluate the effects of silencing MSLN by using short-interfering RNA (siRNA). Mechanistically, these 19–21 oligomers can bind to a specific matching sequence in their target messenger RNAs (mRNAs) and flag them for destruction by a complex referred to as RNA-induced silencing complex (RISC). To achieve this, the sequence of MSLN mRNA was analyzed with specialized software (Qiagen, CA) and sequences for siRNA oligomers were detected. One of these sequences (5′-CTGGACGTCCTAAAGCATAAA-3′) was selected because of its higher degree of specificity against MSLN. The anti-MSLN siRNA oligomer was synthesized (in double-stranded format) and conjugated to Alexa Fluor 488 at the 3′-end of its sense strand (Qiagen, CA). Figure 1A shows the mRNA sequence for human MSLN and the binding site for anti-MSLN siRNA.

In order to investigate the effects of anti-MSLN siRNA on the expression of MSLN, we used the H2373 human mesothelioma cell line. As shown in figure 1B, once electroporated with the anti-MSLN siRNA, the expression of MSLN was notably reduced as early as 24–48 hours post-electroporation as compared with the negative control treated cells. No changes were observed in the expression of β-actin (a house-keeping gene product) after exposure of cells to the anti-MSLN siRNA (figure 1C).

We then decided to test the proliferation rate of cancer cells in such conditions. As is seen in figure 1D, a significant reduction (p<0.005) in the proliferation of mesothelioma cells was observed as early as 48 hours post-electroporation. It is important to note that proliferation rate of control siRNA-treated cells at each time-point was measured and then scaled as 100%. The proliferation rate of anti-MSLN siRNA treated cells was calculated and scaled as a fraction of the control values. The callout panels represent the cell density of the test and control treated populations at indicated times post-electroporation. Interestingly the proliferation rate of siRNA-treated cells started to increase at 72–96 hours post-electroporation. This is due to the transient nature of transfection by electroporation which is the method used in these experiments to introduce anti-MSLN siRNA to cells. In other words, as the time passes, the concentration of siRNA in treated cells would diminish allowing a rebound of proliferation. We have observed such phenomenon in our other studies involving gene specific silencing [26], [27]. Once the same procedure was applied to NIH3T3 cells (void of MSLN), no statistically significant changes in the viability was observed (the siRNA used in this experiment can bind to mouse MSLN mRNA) (figure 1E).

**Figure 2:**
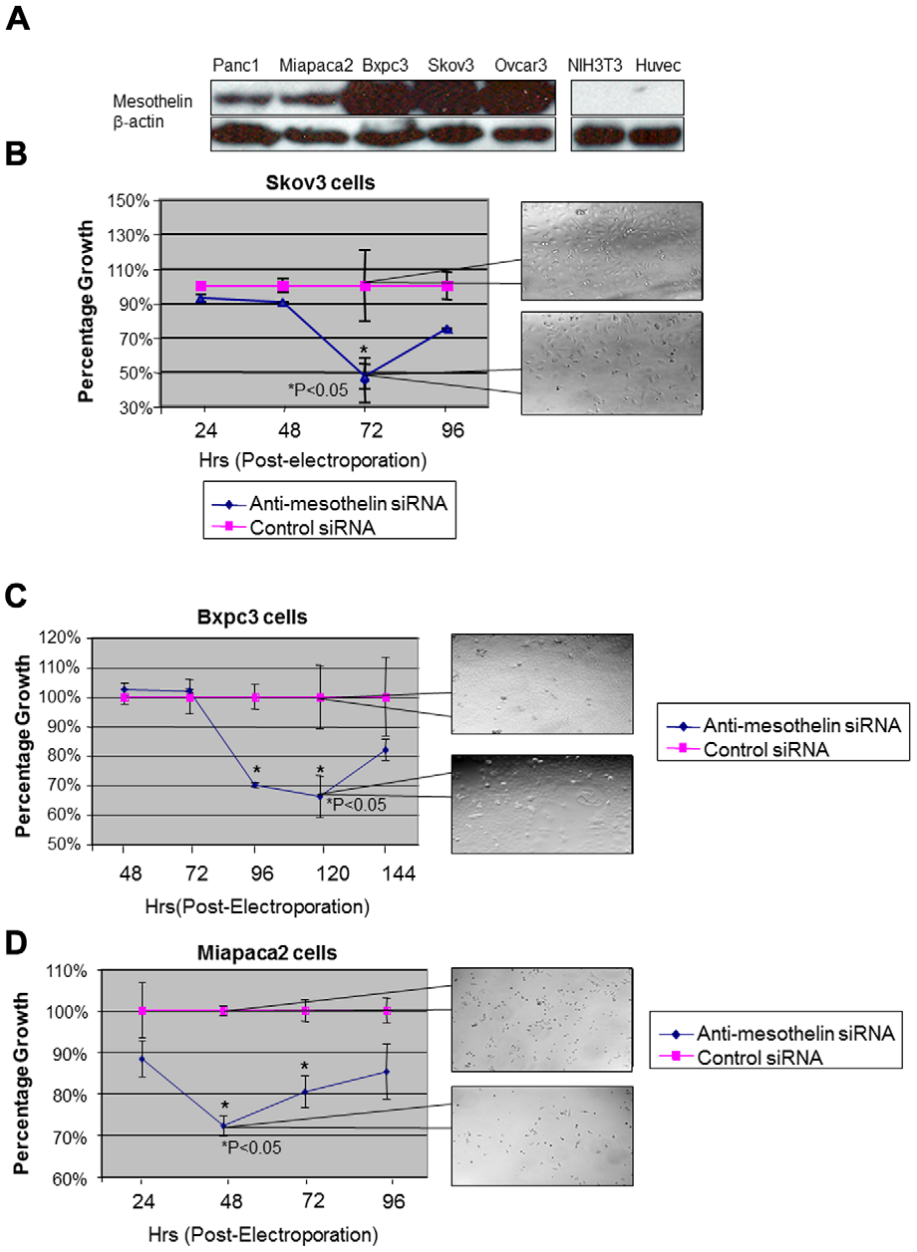
Mesothelin silencing reduces proliferation rate of pancreatic and ovarian cancer cells. (A)Mesothelin protein was detected in pancreatic cancer cell lines, Panc1, Miapaca2 and Bxpc3 and ovarian cancer cells Skov3 and Ovcar3. NIH3T3 and Huvec cells which are void of mesothelin were used to prove the specificity of mesothelin antibody. (B)Skov3 cells had reduced proliferation at day 3 post-electroporation with anti-mesothelin siRNA to about 50% of negative control. Callout panels show the density of cell at each time-point. (C–D)Two pancreatic cancer cell lines, Bxpc3 and Miapaca, were tested for the outcome of silencing mesothelin on their proliferation. In both cases a significant loss of proliferation was observed, however for Bxpc3 the decline initiates at later time points as compared with Miapaca cells. For both cells, once again, a rebound to higher proliferation rates is observed at longer time-points due to the clearance of siRNA from cells. Callout panels show the density of cell at each time-point.

In next step, we decided to test the effects of silencing MSLN in other MSLN-expressing cells including ovarian and pancreatic cancer cell lines and compare such effects with our data about mesothelioma cells. The levels of expression of MSLN in a panel of pancreatic and ovarian cancer cells were tested using western blotting as shown in figure 2A. Miapaca2, BxpC3 and Panc1 (pancreatic cancer cell lines) and Skov3 and Ovcar3 (ovarian cancer cell lines) showed increased levels of MSLN expression while Huvec (Human umbilical vein endothelial cells) and NIH3T3 cells remained negative for MSLN as expected. Electroporation of all above mentioned cancer cell lines resulted in a significant reduction in their viability (figure 2B–2D, results shown for Skov3, BxPC3 and MiapaCa2). Once again a rebound of proliferation due to clearance of siRNA from pancreatic and ovarian cancer cells was observed. The time frame for this rebound was variable among these cell lines due to their relative clearance rate of siRNA.

While all cancer cells showed significant loss of viability upon electroporation with anti-MSLN siRNA, cells with no expression of MSLN such as NIH3T3 did not exhibit significant changes in their proliferation rates once electroporated with anti-MSLN siRNA (targeting mouse MSLN). Therefore, normal cells, with very low or no expression of MSLN would not be affected by this strategy implying the specificity of the biological outcomes of MSLN silencing for malignant cells. Therefore inhibition of MSLN can be considered as a potential strategy for targeting tumors such as mesothelioma, ovarian and pancreatic cancer in a cell specific manner. Recent clinical trials using monoclonal antibodies against MSLN have also been reported to be well-tolerated in patients confirming minimal in-vivo side effects for MSLN targeting strategies [28], [29]. This is of special importance due to the limited levels of mesothelin expression in pleural, pericardial, and peritoneal membranes [30], [31].

**Figure 3:**
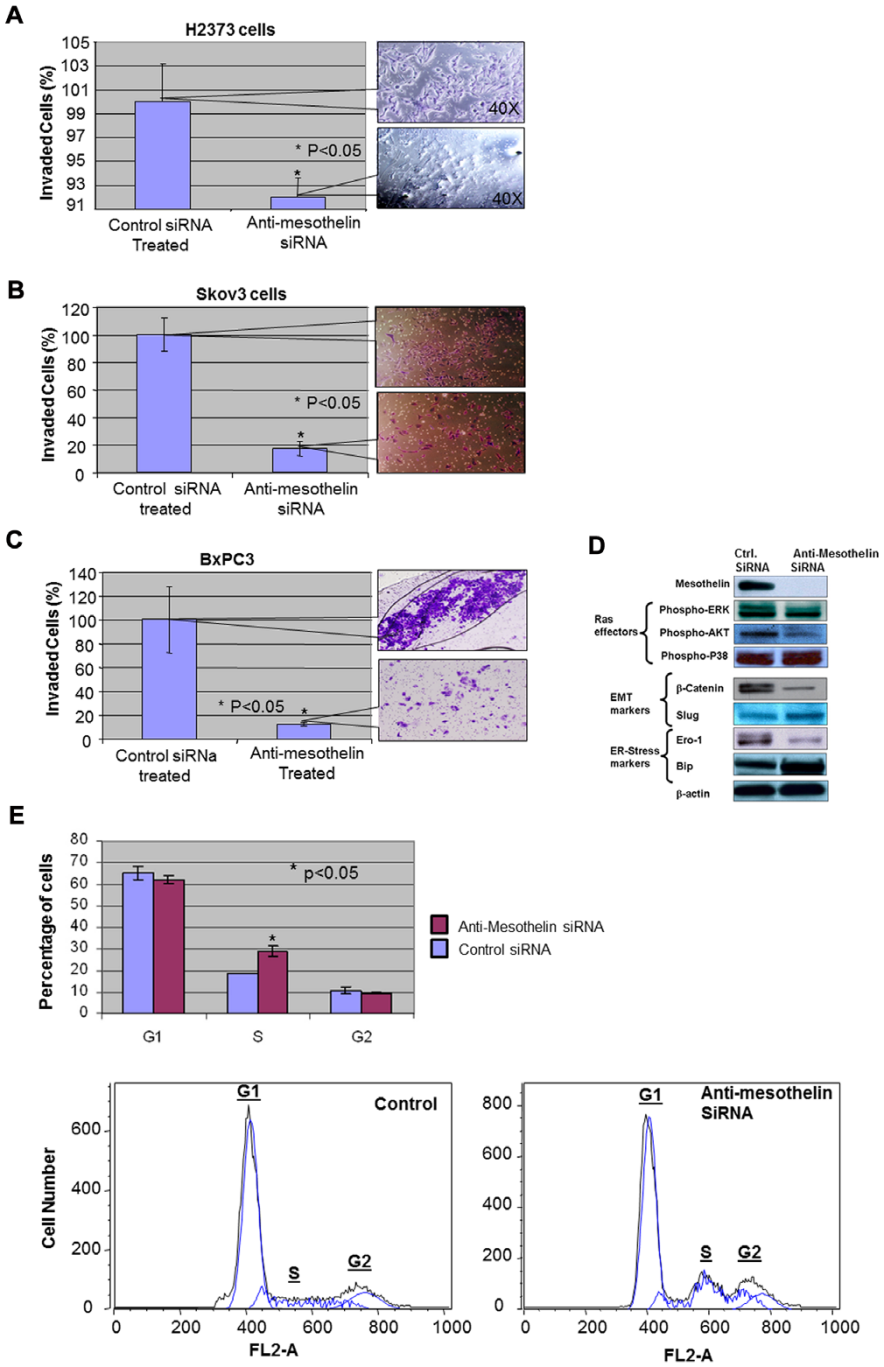
The effects of silencing mesothelin on cancer cell invasiveness, pro-oncogenic cell signaling pathways and cell cycle progression. (A) Once tested in a modified Boyden chamber assay, the invasiveness of H2373 mesothelioma cells is reduced significantly (p<0.05) upon mesothelin silencing. Electroporated cells were introduced to the invasion chambers for this experiment and invaded cells were counted and photographed after 48 hrs. Callout panels represent the density of invaded cells stained with crystal violet. (B–C) Skov3 and Bxpc3 cells both exhibit a significant (p<0.05) decrease in their invasiveness upon mesothelin silencing to values less than 20% of negative control. Callout panels represent the density of invaded cells stained with crystal violet. (D) Silencing mesothelin induces a significant decrease in activation (phosphorylation) of ERK1 (but not ERK2) and phospho-AKT. Additionally, the expression of β-catenin, a known EMT marker, was reduced. Slug, another transcription factor involved in EMT showed a slight increase under this condition. From ER-Stress markers, Ero-1 was decreased while Bip was slightly elevated. (E) Progression of cell cycle is altered by mesothelin silencing mainly by an increase in the percentage of cells in S-phase. The percentage of cells in each phase is shown in upper panel and representative flow cytometry data is offered in the lower panel. G1, S and G2 picks are marked on each graph showing an increase in S phase population of cells.

We were also interested in investigating the corollary of silencing MSLN on the invasiveness of cancer cells. Metastasis as the most devastating outcome of malignancies plays an important role in the pathogenesis of cancer. Therefore, it is a logical step to study the outcome of silencing mesothelin on the capabilities of cancer cells to invade. This is also an important concern considering the invasive nature of malignancies studied in this work. For such purpose we used an in-vitro model based on studying the capabilities of cells to invade through a layer of matrigel as a model for metastasis (modified Boyden chamber assay). We evaluated the invasiveness of H2373, Skov3 and BxPC3 cells once treated with anti-MSLN and control siRNA (figure 3A–3C). In all three cases a significant reduction in invasiveness was observed. The decreased invasiveness of all tested cancer cells is of special clinical relevance due to the high metastatic nature of these diseases.

To this end, we had observed the loss of viability and invasiveness in a range of cancer cells upon silencing of MSLN. In order to somewhat elucidate the molecular mechanism underlying such phenotypic changes we evaluated the activation/expression level of some of the most important signaling proteins involved in neoplastic transformation in H2373 cells (figure 3D). Effector pathways down-stream of proto-oncogene Ras [32], [33], [34] such as activation of ERK1/2 (extracellular signal-related kinase), phosphatidylinositol 3-kinase (PI3K/AKT) and p38 (p38-kinase) were studied for this purpose. We observed that upon MSLN silencing, phospho-ERK1 and phospho-AKT activation levels were profoundly decreased while the levels of phospho-p38 remained unchanged (figure 3D). With involvement of ERK in proliferation and metastasis [35], [36] and also involvement of PI3K/AKT pathway in protection against apoptosis [37], a decrease in the activation of these signaling pathways can explain the anti-proliferative effects of silencing MSLN. However, p38-pathway [38] (involved in stress signaling, apoptosis and senescence) seemed to remain unaltered upon inhibition of MSLN. Since p38-kinase acts as a growth inhibitory pathway, it is conceivable that the capacity of cells to undergo growth inhibition and/or apoptosis (dictated by p38-kinase pathway) remains unaffected by silencing MSLN.

The outcome of siRNA-mediated knockdown of MSLN on epithelial-mesenchymal transition (EMT) [39], [40], a biological program for enhancement of metastatic capabilities, was also investigated by our team. EMT is an important biological step towards achievement of a metastatic phenotype by cancer cells [41]. Beta-Catenin, one of Wnt down-stream signaling molecules and also an important player in EMT, was found to be significantly reduced upon silencing MSLN (figure 3D, the middle panel). Slug, another transcriptional repressor involved in EMT was found to be somewhat increased upon MSLN silencing [42]. With considerations to the role of Slug in repression of E-Cadherin [43] it would be a logical next step to evaluate the expression levels of E-Cadherin upon mesothelin silencing. However, no meaningful change was observed in the levels of E-Cadherin (data not shown). Therefore the incremental increase in the levels of this protein may not positively affect the EMT capabilities of cancer cells once mesothelin is silenced.

We were also interested to investigate the levels of expression of markers involved in the regulation of ER-stress. Stress situations interrupting ER function lead to accumulation of unfolded proteins in the ER which is referred to as ER-Stress [44], [45]. Upon such conditions an integrated panel of signaling pathways becomes activated resulting in the unfolded protein response (UPR) [46], [47]. Following continuation of the UPR response and if the unfolded proteins are not cleared mechanism will be activated to induce cell death. Existence of chronic ER-Stress conditions is becoming increasingly evident in cancer cells [47]. Therefore it would be novel and interesting to see if changes in ER-Stress pathway would contribute to the outcome of MSLN silencing in cancer cells.

ER-residing protein endoplasmic oxidoreductin-1 (Ero1), one of the molecules involves in ER-stress pathway, oxidizes protein disulfide isomerase (PDI), which, in turn, introduces disulfide bands to ER proteins [48]. The significant decrease observed in Ero1 upon MSLN silencing might result in a decreased capacity of cancer cells to fold and clear proteins leading to their eventual death (Figure 3D, lower panel). Also, an increased expression was observed in Bip (Luminal binding protein precursor), a molecular chaperon involved in preventing protein aggregation [49]. Such observation might be indicative of elevated levels of protein unfolding upon MSLN silencing as Bip levels are usually raised in the cell to combat aggregation of unfolded proteins [50].

The last phenotypical feature studied in MSLN-silenced cells was the progression of cell cycle. The major change observed in these cells was a significant increase (∼50%) in the fraction of cells in S-phase portraying a blockade in progression from S to G2 phase (figure 3E).

The current approach for advancing inhibitory RNA therapy to pre-clinical and clinical studies mainly relies on using lentiviruses in order to express and deliver siRNA molecules in a continuous manner [51], [52], [53]. For such purpose, and to produce a translational tool for targeting cancer cells on the basis of inhibition of MSLN, we decided to develop a lentivirus expressing anti-MSLN miRNA. Such tool can be used in future to further evaluate the pre-clinical value of MSLN gene specific silencing as a therapeutic approach.

**Figure 4:**
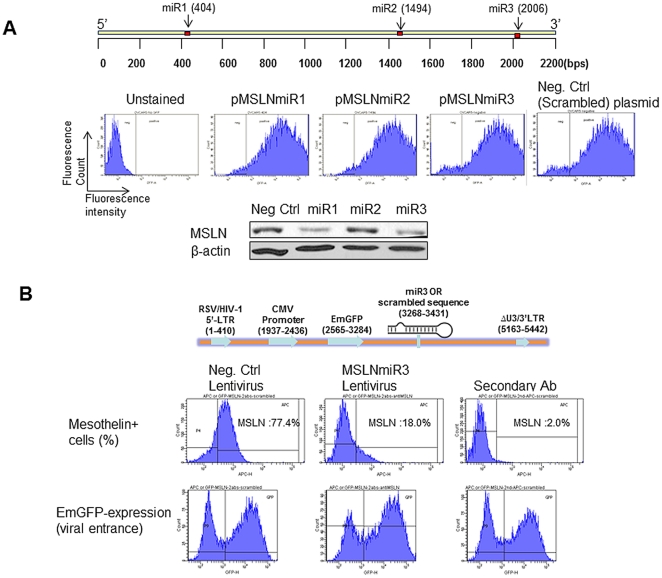
MiRs suppress MSLN expression in human ovarian cancer cells. (A) Upper panel: miRNA mimics (miR) target full length human MSLN gene. The arrow represents the relative position of miRs across MSLN gene sequence. The exact miR sequences are explained in the Experimental Procedure. Middle panel: Expression plasmids encoding scrambled miR or miR targeting MSLN were electroporated (voltage: 1170 V, width: 30 ms, and pulses: 1. Neon Electroporation System, Invitrogen, CA) into human ovarian cancer Ovcar-5 cells and cultured for 48 hrs. EmGFP expression which indicates proper orientation and expression of each miR was determined by FACS. Lower panel: MSLN protein levels were determined by Western blot following electroporation of cells with miRs. (B) Upper panel: Schematic representation of lentiviral genome encoding miR3 against MSLN or scrambled miR. Middle panel: Ovca429 cells were infected by lentiviral particles carrying scrambled miR (Neg. Ctrl) or miR3 against MSLN (MSLNmiR3) at MOI∼30 for 3 days. Expression of MSLN was determined by FACS. The far right panel is infected with negative control virus without staining for MSLN but stained with secondary antibody. Lower panel: The equal expression of EmGFP proves entrance and activity of the negative control and MSLNmiR3 viruses.

As short ribonucleic acid (RNA) molecules (∼22 nucleotides) found in all eukaryotic cells, miRNAs are post-transcriptional regulators which bind to complementary sequences on target mRNA transcripts. This results in translational repression and gene specific silencing [54], [55]. Once a series of three miRNA sequences (miR1, miR2 and miR3, relative positions are shown in figure 4A, upper panel) were selected, each of them was cloned into an expression plasmid (pcDNA6.2-GW/EmGFP, Invitrogen) and electroporated into Ovcar5 cells. All plasmids were efficiently electroporated into the cells (based on GFP expression) (figure 4A, middle panel). Two of the designed miRNAs (miR1 and miR3) out of three silenced MSLN efficiently as was revealed by western blotting (figure 4A, lower panel). Further, miR3 was selected to develop the anti-MSLN lentivirus (named MSLNmiR3). The structure of the MSLNmiR3 lentivirus is shown in figure 4B (upper panel). Ovarian cancer cell line Ovca429 cells, with high levels of MSLN expression, were infected by MSLNmiR3 and its negative control counterpart (encoding scrambled sequence) at MOI∼30. The percentage of MSLN-expressing cells were reduced from ∼77% in cells treated with the negative control virus to 18% in cells treated with MSLNmiR3 (figure 4B, middle panel). For both viruses, the viral entrance was almost equal as was proven by EmGFP expression (figure 4B, lower panel).

**Figure 5:**
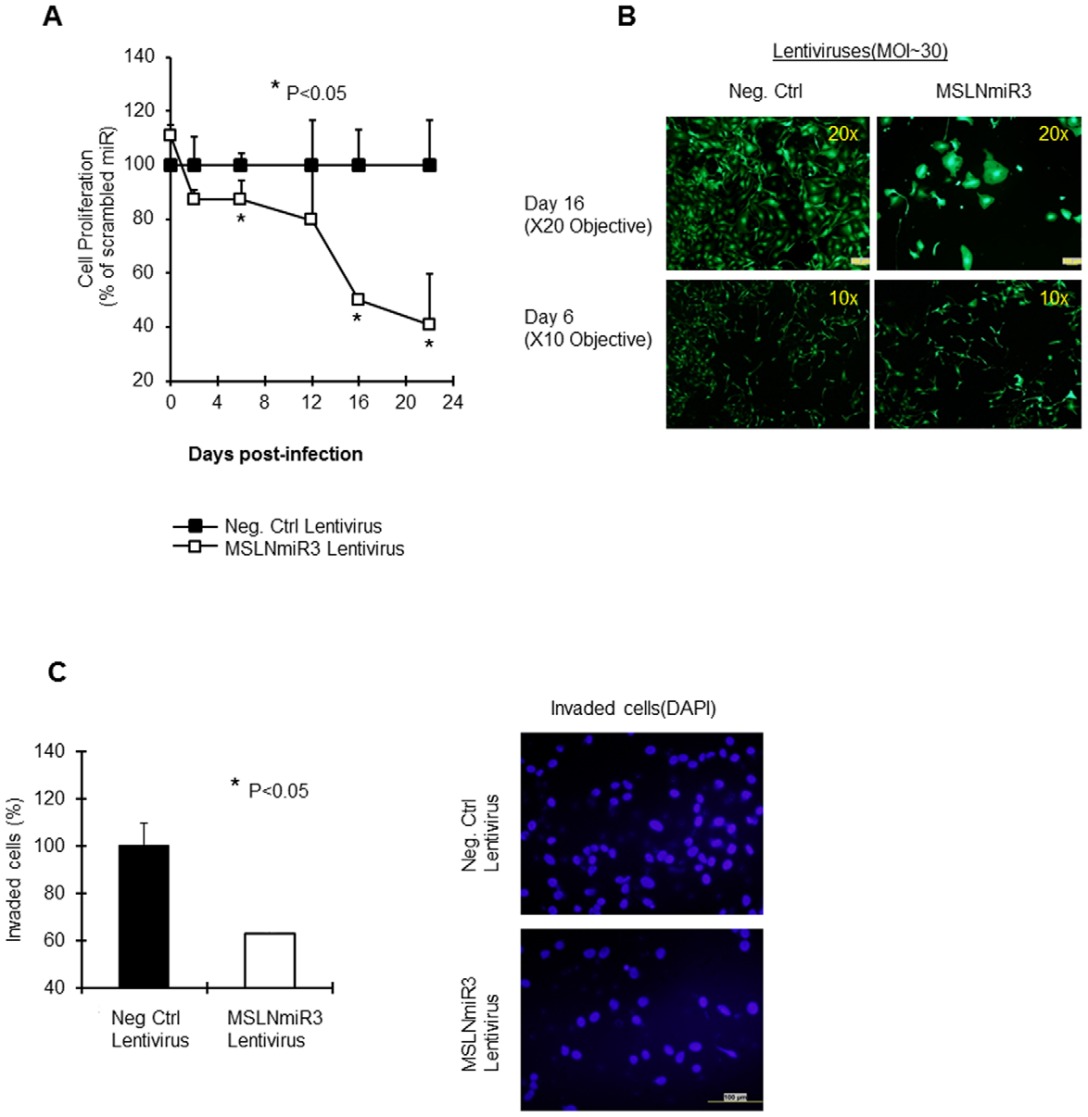
Silencing MSLN confers inhibited cell proliferation and invasion. (A) Ovca429 cell were infected with MSLNmiR3 or Negative Control virus at MOI∼30. Cell proliferation was assessed at the indicated time-points. (B) Photographs of Ovca429 cells expressing EmGFP post-infection at the indicated time-points. 10× objective was used for taking pictures at day 10 and 20× was used at day 20 in order to focus on morphological changes of cells. (C) Left panel: Three-day post infection, Ovca429 cells were transferred and cultured in Boyden assay invasion chambers for 21 hrs. Cells were washed, fixed, and cell nuclei were stained by DAPI. Representative fields from each group were then counted for the number of nuclei and averages were calculated and compared (p<0.05). Numbers of cells invaded for the negative control virus was considered as 100%. Right panel: Photographs of invaded cells nuclei in MSLNmiR3 and negative control virus treated groups. The bar shows 100 µM in length.

In next step, we evaluated the viability of ovarian cancer cells once treated with this virus. The viability of Ovca429 cells upon infection with MSLNmiR3 (MOI∼30) showed about 15% decreases at day-6 post-infection and about 50%, 60% reduction at day-16 and day-22, respectively (figure 5A). This is achieved by treatment with a single dose of the virus. Figure 5B shows the morphology of cells treated with MSLNmiR3 or the negative control virus at day-16 (20× objectives) and day-6 (10× objectives). An interesting phenomenon was the enlargement of remaining cells upon exposure to MSLNmiR3 lentivirus. This might entail a role for MSLN in machinery involved in the cell morphology, polarity and cytoskeletal reorganization which influences invasiveness of cancer cells although the field needs more studies in this regards [41], [56], [57]. Additionally, the resemblance in the biological outcome of silencing mesothelin by two distinct agents, siRNA and miRNA, reduces the possibility of off-target events and involvement of any player other than MSLN in this scenario. Also, the rate of loss of viability upon targeting cancer cells with anti-mesothelin lentivirus is notably slower as compared to silencing mesothelin by siRNA electroporation. This is due to the kinetics of the expression of miRNA from the context of lentiviral genome which happens at a much slower rate in comparison to the rapid influx of siRNA to cytoplasm achieved by siRNA electroporation.

Finally, direct study of cell invasiveness in a modified Boyden chamber assay also showed a close to 40% reduction in the metastatic capability of Ovca429 cells at day-2 post-infection. Such information reveals a significant role for mesothelin in influencing the metastatic capability of cancer cells. The work, therefore, provides clues to the mechanisms involved in the role of MSLN in cancer cell's proliferative capabilities, invasion (through EMT marker changes), ER-Stress signaling and phenotypical changes.

### Conclusion and Future Directions

The biological function of MSLN is not well-understood. This study delineates functional role of MSLN in cancer cell survival and possible signaling pathways that may confer MSLN action in a number of distinct malignancies. This is indeed of significant importance because cancer models pursued in this report (mesothelioma, pancreatic and ovarian cancers) seem to collectively follow the outcome of elimination of MSLN as a biological turn-point.

It is also important to take notice of the level “cancer cell addiction” to the expression of this differentiation antigen for the proper maintenance of its biological functions. Our data in all studied cancer models show that a significant loss of viability follows the loss of MSLN expression. In light of this, MSLN can be rendered as suitable candidate for drug therapy with focus on finding novel small molecule inhibitors which can bind and inhibit MSLN. Our data in regards to cell signaling changes upon MSLN silencing are useful for developing evaluation assays for studying biochemical function of such anti-MSLN compounds. Once such compounds with inhibitory function against MSLN are available, their effect on the relationship between MSLN and CA125 can be studied. Our team is involved in such drug discovery efforts as well and has found a few compounds with enhanced cytotoxicity against MSLN expressing cancer cells (unpublished data).

The other tool provided in our work, the anti-MSLN lentivirus (MSLNmiR3), can also serve as a translational tool for the gene therapy of MSLN expressing tumors. Animal experiments are under way for evaluating the efficiency of this virus in causing tumor regression in-vivo. Altogether, our findings revealed MSLN as a potential target for achieving better understanding of the biology of a number of human malignancies as well as designing novel therapeutic strategies for cancer therapy.
